# Investigating the Spatial Dimension of Food Access

**DOI:** 10.3390/ijerph14080866

**Published:** 2017-08-02

**Authors:** Jackie Yenerall, Wen You, Jennie Hill

**Affiliations:** 1 Office of Health Policy, Tennessee Department of Health, Nashville, TN 37208, USA; 2 Department of Agricultural and Applied Economics, Virginia Tech, Blacksburg, VA 24060, USA; wenyou@vt.edu; 3 Department of Epidemiology, University of Nebraska Medical Center, Omaha, NE 68198, USA; jennie.hill@unmc.edu

**Keywords:** food access, grocery store accessibility, obesity, cluster analysis

## Abstract

The purpose of this article is to investigate the sensitivity of food access models to a dataset’s spatial distribution and the empirical definition of food access, which contributes to understanding the mixed findings of previous studies. Data was collected in the Dan River Region in the United States using a telephone survey for individual-level variables (*n* = 784) and a store audit for the location of food retailers and grocery store quality. Spatial scanning statistics assessed the spatial distribution of obesity and detected a cluster of grocery stores overlapping with a cluster of obesity centered on a grocery store suggesting that living closer to a grocery store increased the likelihood of obesity. Logistic regression further examined this relationship while controlling for demographic and other food environment variables. Similar to the cluster analysis results, increased distance to a grocery store significantly decreased the likelihood of obesity in the urban subsample (average marginal effects, AME = −0.09, *p*-value = 0.02). However, controlling for grocery store quality nullified these results (AME = −0.12, *p*-value = 0.354). Our findings suggest that measuring grocery store accessibility as the distance to the nearest grocery store captures variability in the spatial distribution of the health outcome of interest that may not reflect a causal relationship between the food environment and health.

## 1. Introduction

### 1.1. Background

In recent years the built environment, and more specifically the food environment, has received increasing attention as a possible explanation for the obesity epidemic [1,2,3,4,5]. The food environment is a broad term describing all places where food is available for purchase or for consumption, including grocery stores, supercenters, corner stores, and all types of restaurants [6,7,8]. Access has been proposed as a potential mechanism through which the food environment affects individual health outcomes, particularly obesity [1,2,9,10,11]. Access is defined by five dimensions: availability, accessibility, affordability, acceptability, and accommodation [10]. Each dimension captures an attribute of the food environment that may impede an individual’s ability to acquire an adequate supply of food. Availability and accessibility capture the geographical ease with which individuals can access food retailers. Affordability captures objective and subjective measures of cost, and the remaining two dimensions, acceptability and accommodation, reflect perceptions and cultural norms related to the food environment [6,12].

Among the five dimensions the two geographic components, availability and accessibility continue to be popular choices for study and, given their spatial dimension, allow for additional empirical testing using geographic information systems and various spatial analyses [9,10,13,14]. Accessibility is commonly measured as the distance from the household to the nearest food retailer of a specific type, while availability is measured by the number or density of retailers within a certain radius of a household. Supermarkets and grocery stores are generally conceptualized as indicating a healthy food environment, while fast food restaurants and convenience stores are conceptualized as indicating an unhealthy food environment [13,15,16]. The underlying hypothesis in food access papers using the accessibility or availability dimensions is that healthy food access, as measured by shorter distances to or higher densities of healthy food retailers (i.e., supermarkets and grocery stores) should be correlated with a decreased likelihood of obesity [17]. However, results from the empirical literature examining the effect of access to healthy food on obesity have been mixed, and even counter-intuitive, and when significant relationships have been found, the magnitude is often small [6,11,12,13,17,18]. Furthermore, some evaluations that have assessed the impact of the opening of a new healthy food retailer have found no effect on individual-level behavior or health outcomes [14,19,20]. However, Cummins et al (2014) found a significant improvement on perceptions of food accessibility [14]. Investigating the spatial dimension of food access using methods from spatial statistics may provide additional insights into these mixed findings.

### 1.2. Spatial Dimension and Empirical Challenges

The spatial dimension of accessibility refers to the additional layer of information that is available in a spatially sensitive dataset (i.e., datasets with the geographic location of the observations). Specifically, in accessibility models the spatial dimension is important when the distance between the home and the nearest retailer is used to generate the primary covariate of interest: accessibility. This additional source of variation from the spatial distribution creates three empirical considerations, which are important to consider when disentangling the heterogeneity caused by a violation in the assumption of independently and identically distributed error terms from the true differences in the outcome.

First, statistical methods commonly used to analyze food access models (such as linear or logistic regressions) assume that the observations are distributed independently and identically. In spatial datasets, this assumption is known as complete spatial randomness (CSR) [21]. Satisfying this assumption is required to estimate unbiased standard errors. However, in spatial datasets it is often violated because observations that are close together, based on a measure of geographical distance, are more likely to be similar than observations that are far apart geographically [21]. This is a violation of the independence portion of the assumption and can bias inference if not addressed [3,4,5,21,22]. Global spatial statistics can test for variation in the spatial distribution that would violate the CSR assumption, and, in the past, food access research has addressed this challenge by utilizing cluster robust standard errors, spatial lag models, and other specialized models that account for spatial autocorrelation in the estimation of the variance-covariance matrix [21,23,24,25,26].

Second, the underlying hypothesis in the accessibility dimension is that the distance from a retailer affects the likelihood of being obese, which suggests the existence of local spatial variation in the data, and could result in spatial heterogeneity. Spatial heterogeneity is a concern because it confounds the interpretation of the food access parameter. Linear or logistic regressions assume that food access parameters are constant over space; spatial heterogeneity violates this assumption and instead indicates that a relationship varies over space [27,28]. However, spatial heterogeneity can also be viewed as informative because it may identify areas in a geographic study where the relationship between the food environment and obesity is strongest and may be best suited for an intervention.

Spatial heterogeneity has been addressed in previous research using specialized models known as geographically-weighted regression [27,28]. Chi et al. (2014) found evidence of spatial heterogeneity in a model used to estimate the relationship between obesity and the food environment at a national scale using data from the United States [27], while Fraser et al. (2012) found evidence in a model estimating the relationship between the food environment and consumption using data from the Avon region in the United Kingdom [28].

Scanning statistics can be used to detect local sources of spatial variation that may indicate heterogeneity by identifying clusters using either areal data, which are collected at an aggregated geographic level such as the census tract, or point data, which are collected at the occurrence of an outcome [29]. By assuming the data is distributed with CSR, scanning statistics can calculate the expected number of outcomes and compare this to the observed number of outcomes [29]. A cluster exists in the case that the observed number of outcomes is greater than the expected number of outcomes [29]. These methods have been used in spatial epidemiology to detect hot spots or create disease maps, which are used to understand the spatial distribution of a health outcome of interest and identify variables to explain this spatial pattern [21,30]. They have also been used in the food environment literature to examine the spatial distribution of food retailers and identify food deserts defined as a cluster in which the number of healthy food retailers is lower than expected [31,32].

The final empirical consideration presented by the spatial dimension concerns the potential for bias in the estimation of the effect of accessibility on health outcomes. While there are many ways that bias can arise in the estimation process, this discussion focuses on two that are related to the spatial dimension of accessibility: self-selection and measurement error. First, because individuals do not choose where to live randomly, it is possible individuals with similar health outcomes will share housing location preferences that may be related to the food environment [25]. This self-selection undermines a causal interpretation unless the endogeneity of the food access parameter is addressed. Second, it is often assumed in accessibility models that quality is captured by retailer types (example: grocery stores vs. fast food restaurants), but this assumes that all stores within a given food retailer category are homogenous in quality because the distance to all stores within a food retailer type is weighted equally [15,33,34,35,36]. However, food environment research that has addressed the non-geographic dimensions, including affordability, acceptability and accommodation, has identified significant differences in quality between food retailers within a retailer type, such as supermarkets [37,38,39]. Thus, this assumption in accessibility models may result in measurement error bias if there is considerable quality variation within food retailer categories and if these variations correlate with the health outcome of interest.

### 1.3. Objectives

The purpose of this article is to test the sensitivity of food accessibility models to the presence of local spatial variation, especially as it relates to the empirical considerations discussed above. This goal is accomplished through two objectives. First, local spatial variation is detected using a combination of spatial methodology and a logistic regression that uses grocery store accessibility as the covariate of interest. Scanning statistics are used to identify the presence of local spatial variation suggested by accessibility. If the interpretation of the scanning statistic matches that of the regression model, this serves as a first indicator for the presence of spatial heterogeneity. This is because the regression model assumes the accessibility parameter is constant over the entire geographic region, while the scanning statistic is designed to detect local spatial variation that is not constant over the geographic region.

Second, to further test for the possibility that the regression results are sensitive to local sources of spatial variation, a measure of retailer specific quality is used to create a quality weighted measure of accessibility. A quality-weighted measure of accessibility is used to address potential measurement bias in the accessibility variable that arises when the true relationship between accessibility and obesity is more complicated than distance alone. If the relationship detected in the original regression is robust to the quality adjustment, then it leads to the conclusion that the relationship detected in the model is more likely to reflect a behavioral response to the environment. However, if the results are not robust then it may indicate that the findings are sensitive to the local spatial variation, which may be a consequence of spatial heterogeneity.

Addressing these objectives requires data that include health outcomes for and the geographical location of individuals, the location of food retailers, and an objective measure of the quality of those food retailers. This study leveraged a unique dataset that includes a validated measure of grocery store quality (Nutrition Environment Measures Survey (NEMS-S) [40], individual-level obesity outcomes from a regional surveillance study, and other spatial elements.

## 2. Materials and Methods

### 2.1. Data Source

The data for this study came from the Dan River Partnership for a Healthy Community (DRPHC), a community academic partnership in the Dan River Region (DRR) [41]. The DRR consists of three counties that are classified as rural by the USDA Rural Urban Community Area Codes (RUCA). However, the study area also contains a mid-sized regional city of approximately 43,000 residents and a small city of approximately 10,000 residents. Both cities are classified as urban clusters using census definitions [42,43]. Because the dataset consists of both rural counties and urban cities within close geographical proximity, analyses were conducted with observations from urban and rural areas pooled and separately.

The data analyzed in this paper were collected using two methods: a telephone survey that collected individual-level data, described in Section 2.1.1, and an audit of food retailers in the DRR that collected the food environment variables described in Section 2.1.2. Physical addresses were collected and used to geocode the location of retailers and the telephone survey respondent’s household address; this resulted in a point-referenced dataset.

#### 2.1.1. Individual-Level Data

Individual-level data were collected in the region via a telephone survey, which sampled listed and unlisted telephone numbers along with both landlines and cell phones [42]. Random proportional sampling based on the population of the three counties and two cities was used to create a geographically representative sample [42]. The response rate for the survey was 77%, resulting in a final sample size *n* = 784 [42]. The survey was modeled after the Virginia and National Behavioral Risk Factor Surveillance System (BRFSS) surveys for 2011 and was conducted by a professional survey unit [42]. Responses from this survey were used to create several individual-level variables used in the analysis including: self-reported height and weight, sex, age, self-reported measures of income, highest level of education attained, and employment status.

Self-reported height and weight were used to calculate a continuous measure of body mass index (BMI) based on the following formula: $\mathrm{BMI}=\left(\frac{\mathrm{weight}\left(\mathrm{kg}\right)}{\mathrm{height}\;{\left(m\right)}^{2}}\right)$. Continuous BMI values were converted to a dichotomous variable for obesity that is equal to one for values of BMI greater than or equal to 30, and zero for all others.

#### 2.1.2. Food Environment Data

Store-level data were collected in two stages, which are described in greater detail in Chau et al. (2013) [44]. In the first stage, food outlets in the DRR were identified using a database of active permits to sell food, which was provided by the Virginia Department of Health. Outlets that served a worksite or school, or did not serve the public, were excluded. This list was divided into stores and restaurants and was then categorized by the NEMS classifications [40,45]. This included two main categories for food stores, grocery stores, and convenience stores, and a third category for fast food restaurants that are used in this study [44]. In the second stage, food outlets were evaluated using the Nutrition Environment Measures Survey (NEMS) surveys [44]. The purpose of the NEMS is to assess the food environment as it relates to factors that would affect food choice: price, availability, and quality [40,45]. Specifically, this paper utilizes the results from the NEMS-S for grocery stores.

NEMS-S uses three dimensions: availability of more healthful or recommended choices, quality of produce, and prices to evaluate the quality of store [40]. Availability was assessed for ten food categories: fruits, vegetables, milk, ground beef, hot dogs, frozen dinners, baked goods, beverages, whole grain, and baked chips. The quality dimension was based on a visual inspection of fruits and vegetables for bruising, looking old, over ripening, or spotting. Price was assessed using the non-sale price per pound of fruits and vegetables, and the relative price of the healthier versus standard food options (example: skim versus whole milk).

Data from these store audits were used to create several variables related to the food environment for this study. The NEMS store variable used in analysis was calculated by summing the scores across the three aforementioned dimensions of NEMS-S. Higher NEMS scores indicate a better quality for that store. Other variables calculated in ArcGIS v10.2 (ESRI, Redlands, CA, USA) were: network distance to the nearest grocery store and its NEMS score, network distance to the nearest fast food restaurant, and noxious (fast food and convenience store) retailer availability. Noxious retailer availability was measured as the count of retailers within a 10-mile radius of an individual’s home for individuals located in rural areas and a 1-mile radius for individuals located in urban areas [15].

### 2.2. Analytic Plan

For the first objective of this paper, which is to identify sources of local spatial variation that may indicate spatial heterogeneity, the analytical plan will include the use of unconditional spatial statistics and conditional logistic regression.

Unconditional spatial statistics are used to assess the spatial distribution of data for a given geographic area of interest. Prior to using scanning statistics to identify local sources of variation, it is necessary to use global spatial statistics to test for patterns that will affect the specification of the conditional logistic regression [21]. Nearest neighbor is a global spatial statistic used to identify violations of complete spatial randomness (CSR) for the full geographic area of interest. The null hypothesis assumes that the data is distributed with CSR, meaning that observations are distributed at random and uniformly over the study area. Rejecting the null indicates the presence of a clustered or regular pattern [21]. A clustered pattern indicates that cluster robust standard errors are necessary in the conditional logistic regression to minimize the risk of bias in the estimation of the standard errors.

Scanning statistics are used to assess local spatial variation by identifying clusters within a geographic area. Scanning statistics assume the outcome follows either a Poisson or Bernoulli distribution [29]. The Poisson distribution is used to detect clusters in areal data (example: obesity prevalence at the census tract level), while the Bernoulli distribution detects clusters in point-referenced data (example: individual outcomes). Specifically, the scanning statistic with the Bernoulli distribution detects clusters of a certain outcome, known as cases. For obesity, individuals who are obese would be identified as cases and the scanning statistic would detect areas in which a higher than expected number of obese outcomes occurred. This statistic can also be used to detect clusters relative to a fixed location, such as the location of a food retailer [29].

A likelihood ratio test is used to detect statistically significant clusters, also known as windows, for both the Bernoulli and Poisson forms of the scanning statistics [29]. The likelihood function used in the Poisson version of this test is given in Formula (1), while the Bernoulli version is given in Formula (2).

(1) $$\;L\left(W,p,q\right)=\frac{e^{-pµ\left(W\right)-q(µ\left(G\right)-µ\left(W\right)0}}{\;n_{G}!}p^{n_{w}}q^{(n_{g}-n_{w)}}\underset{x_{i}}{\prod }\mu \left(x_{i}\right)\;$$

(2) $$\;\left(L,W,p,q\right)=p^{n_{w}}{\left(1-p\right)}^{µ\left(W\right)-n_{w}}q^{n_{G}-n_{w}}{\left(1-q\right)}^{\left(µ\left(G\right)-µ\left(W\right)\right)-\left(n_{G}-n_{W}\right)}\;$$

where *W* is a window, defined by a foci that identifies the center of the window, while the radius determining the size of the window is variable; *p* is the probability of an outcome in the window, *q* is the probability of an outcome outside the window, *n_w_* is the number of cases in a window, µ(*W*) is the total number of observations in the window, and *n_G_* is the total number of observations in the study area.

The null hypothesis is that the probability of an individual being located within a window (denoted as *p*) is equal to the probability of being located outside the window (denoted as *q*) [30]. The alternative hypothesis is that *p* > *q* (i.e., the window represents a cluster). The window with the largest likelihood value is identified. Its likelihood value becomes the numerator of the likelihood ratio test, and the denominator is the likelihood value associated with the null hypothesis. SaTScan version 9.4 (Boston, MA, USA) was used to estimate the scanning statistics and perform the likelihood ratio tests. Results report the number and statistical significance of clusters identified and the associated length of the radius for statistically significant clusters.

For objective one, both forms of the scanning statistics will be used. First, the Poisson distribution is used to detect clusters of grocery stores at the block group level, which indicates a healthy food environment. The Bernoulli distribution is then used to detect clusters at the individual level, and specifically for the obesity outcome relative to grocery stores, by using the locations of grocery stores as the foci for the scanning statistic. Jointly, these statistics can be used to identify local variation spatial that suggests living closer to a grocery store increases the likelihood of obesity.

A conditional logistic regression is then used to represent a typical food access model to estimate the relationship between accessibility and obesity. Covariates are included to control for the effect of sex, race, age, highest level of education, income, and employment status. Cluster robust standard errors at the census tract level are used to account for clustering. The model is estimated for the full geography and separately for rural and urban sub-samples.

Three versions of this model are used to support the objectives of this paper, and all are estimated in STATA version 14 (StataCorp, College Station, TX, USA). The first model supports objective one and uses a typical empirical specification of grocery store accessibility in which accessibility is measured as the distance from the home to the nearest grocery store. If grocery store accessibility is statistically significant and negative it indicates the model is sensitive to the local spatial variation detected by the scanning statistics and suggests the presence of spatial heterogeneity.

Results from the second and third model support objective two, which addresses potential measurement error bias in the accessibility parameter by using quality-weighted grocery store accessibility (QWGA) in place of the typical measure of accessibility. The third model also includes a noxious retailer availability variable to control for the effect of a poor food environment. 

QWGA uses both the distance to the nearest grocery store and the NEMS score of the nearest grocery store to create a quality weighted measure of accessibility. More specifically, QWGA uses the rank of the NEMS score that is created by ordering the NEMS score of all grocery stores in the sample (i.e., stores that correspond to a nearest grocery store of an individual in the survey) from lowest to highest to reflect increasing quality. Then, the typical measure of accessibility is divided by the rank of the nearest grocery store. This creates a measure that is sensitive to changes in distance and quality, but maintains the same interpretation as the typical measure in which smaller values correspond with better accessibility. The formula for QWGA is:

(3) $$QWGA=\frac{Distance\;from\;household\;to\;nearest\;grocery\;store}{NEMS\;rank\;of\;nearest\;grocery\;store}$$

To illustrate the value of the *QWGA* variable, consider the following example using a sample of four households (A,B,C,D) and corresponding food environment variables as outlined in Table 1.

In Table 1 the households are listed in order of decreasing grocery store accessibility (i.e., longer distance to a grocery) but increasing grocery store quality (i.e., larger NEMS score). Using distance alone would lead to the conclusion that household D has the poorest accessibility, while household A has the best accessibility. However, this conclusion does not take into account the fact that the quality of nearest grocery store for household D is six times higher than that for household A. QWGA is sensitive to both of these pieces of information because it weights the distance to the nearest grocery store by dividing it by the NEMS rank.

The significance of this distinction is also apparent when comparing household B and C. For both households the distance to the nearest store is the same. Thus, a typical model of accessibility would treat these households equally, despite the NEMS score being twice as high for household C. Using QWGA gives a better measure of accessibility by reflecting that for the same distance traveled a higher quality grocery store indicates better accessibility.

## 3. Results

### 3.1. Summary Statistics

Table 2 contains the individual-level summary statistics for the full sample, the geographic subgroups, and results from *t*-tests to compare the differences between the rural and urban subsamples. Urban residents had a higher mean BMI (M = 0.25 vs. M = 28.21, *p*-value < 0.001) and a higher prevalence of obesity (M = 43.87% vs. 34.31%, *p*-value = 0.007).

The urban sample also contained a higher percentage of females (M = 80% vs. M = 73.69%, *p*-value = 0.04) and African Americans (M = 58.41%, vs. M = 19.88%, *p*-value < 0.001). Additionally, this sample tended to be younger (M = 51.69 vs. M = 58.64, *p*-value < 0.001). There are fewer socioeconomic differences, but significant differences include the percentage of individuals who are retired (M = 23.81% vs. M = 37.35%, *p*-value < 0.001), with an income less than $20,000 USD (M = 65.36% vs. M = 32.07%, *p*-value < 0.001), with an income between $20,000 USD and $50,000 USD (M = 19.29% vs. M = 43.23%, *p*-value < 0.001), and with an income greater than $50,000 USD (M = 15.36%, M = 24.70%, *p*-value = 0.003).

Table 3 contains summary statistics for the food environment variables for the full sample, the geographic subgroups, and results from *t*-tests to compare the differences between the rural and urban subsamples. Significant differences were found between all variables, which included: further distance to the nearest grocery store (i.e., grocery store accessibility) for rural residents (M = 5.24 vs. M = 1.19, *p*-value < 0.001) and shorter distance to the nearest fast food retailer for urban residents (M = 0.96 vs. M = 3.98, *p*-value < 0.001). Additionally, there are significant differences in the NEMS score of the nearest grocery store (M = 23.10 vs. M = 20.40, *p*-value < 0.001), noxious store availability (M = 8.92 vs. M = 57.99, *p*-value < 0.001), and QWGA (M = 0.10 vs. 0.73, *p*-value < 0.001). These results suggest that even in a limited geographic area, the differences between rural and urban food environments are sufficiently different to warrant separate analyses.

### 3.2. Unconditional Spatial Statistics

Results from this section correspond to the first objective of the paper, which is to investigate potential sources of local spatial variation that are suggestive of spatial heterogeneity using scanning statistics and a conditional logistic regression. Results for the logistic regression will be discussed in the next section.

Table 4 contains the results for the scanning statistics. The Poisson distribution was used to identify areas with relatively healthy food access, defined by a higher than expected number of grocery stores within a block group, or unhealthy food access, defined by a lower than expected number of grocery stores within a block group. The table includes the total number of clusters identified in the region (i.e., all windows with *p* > *q*) and the length of the radius for any statistically significant clusters. Only one significant cluster was identified in the full geography (*p*-value < 0.001). This cluster contained a higher than expected number of grocery stores, which indicates an area of healthy food access. Using the coordinates of the center of the radius for the cluster, it was determined that the cluster was located in the mid-sized regional city.

The Bernoulli scanning statistic was used to detect clusters of obese individuals with grocery store foci. The presence of such a cluster would suggest that living closer to a grocery store increases the likelihood of obesity. Results are also presented in Table 4. Only one significant cluster of obese individuals with grocery store foci was found (*p*-value < 0.001). The radius of this cluster is 1.48 miles, putting it well within the bounds of the 10-mile radius used by the United States Department of Agriculture (USDA) to define good access within more rural areas; although it is slightly larger than the 1-mile radius used for urban areas [15]. This statistic indicates there are a disproportionately high numbers of obese individuals within a region that would otherwise be characterized as having good access to a grocery store.

**Figure 1:**
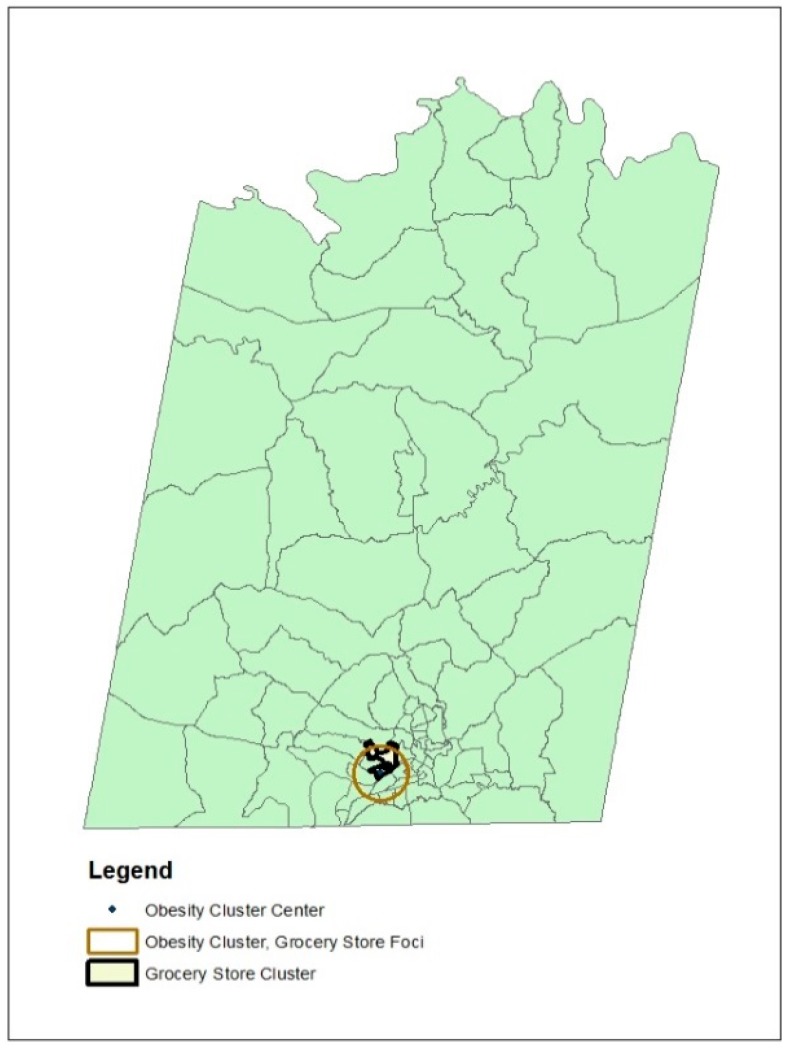
Overlap of grocery store and obesity clusters identified from spatial scanning statistics.

Finally, the results from the Poisson and Bernoulli versions of the scanning statistic were overlaid to detect possible overlap between the cluster of healthy food access (as represented by the higher than expected cluster of grocery stores) and poor health outcomes (indicated by clusters of obesity relative to grocery stores). Figure 1 shows the overlap between the grocery store cluster, which is outline by a dark black border, and the obesity cluster, which is outlined by a lighter grey border. The dot within this block group shows the location of the grocery store, which was the foci of the cluster of obese individuals. This overlap occurred within the mid-sized regional city contained in the sample.

Scanning statistics revealed an overlap between good access to healthy food and a cluster of obesity, which would be a counterintuitive finding. However, these are unconditional statistics and the relationship detected from this section could be moderated by other demographic and socioeconomic factors or other elements of the food environment. Additionally, the scanning statistics rely only upon the distribution of grocery stores across space and are unable to account for variation in the quality between grocery stores detected in summary statistics contained in Table 3.

Finally, the nearest neighbor statistic for the sample was 0.54 (*p*-value < 0.001), indicating clustering. This finding informs the use of cluster robust standard errors in the conditional logistic regression.

### 3.3. Conditional Logistic Regression

Table 5 contains the results from the conditional logistic regression where results from the control variables are suppressed. Given the non-linear nature of a logistic model the average marginal effects (AME) are reported.

The results from Model 1 are used in conjunction with the results from the scanning statistics discussed in the previous section to detect potential spatial heterogeneity related to local spatial variation. In the typical accessibility model, Model 1, the average marginal effect (AME) of grocery store accessibility is only significant in the city subsample (AME = −0.09, *p*-value = 0.02). The sign indicates that on average increasing the distance to the nearest grocery store is associated with a decrease in the probability of being obese, which is counterintuitive. However, it is also consistent with results from the scanning statistics that found a cluster of obese individuals relative to a grocery store in the regional city. Given that the accessibility parameter is significant only in the urban subsample it suggests the presence of spatial heterogeneity.

Models 2 and 3 used the QWGA to address the second objective. In Model 2 the QWGA is not significant in any geographic area, including the city subsample (AME = −0.12, *p*-value = 0.354). This implies that controlling for heterogeneous quality of grocery stores is an important consideration and may suggest a more nuanced relationship than can be detected by geographic distance alone. Expanding the utilized definition of food environment to include a measure of unhealthy food access, noxious food availability, in Model 3 had no effect on the conclusions, as all food environment variables were statistically insignificant.

## 4. Discussion

Given the changes in the local food environment and obesity over the past 30 years, researchers have sought to identify what, if any, relationship exists between the two. While research has consistently identified areas of poor access, findings on the relationship between the geographic dimensions of access, accessibility and availability, and obesity have been mixed [6,11,13,23,46]. Investigating the spatial dimension of accessibility may provide some insight into these mixed findings. This paper proposes to investigate this possibility through two objectives and by using data from the Dan River Region (DRR) in the United States. First, spatial scanning statistics were used to investigate local spatial variation that may indicate spatial heterogeneity in regression analysis. Second, a quality-weighted measure of grocery store accessibility (QWGA) was used to address potential measurement error bias in the accessibility parameter.

The first objective is motivated by the underlying hypothesis in the accessibility dimension that implies the distance from a food retailer should affect the likelihood of obesity. Logistic regressions are generally used to test for this relationship, however, this model assumes that the relationship between distance and obesity is constant over space. This paper uses scanning statistics to test for the presence of local spatial variation that would violate the assumption in the logistic regression. If the interpretation of the scanning statistics matches that of the logistic regression, it could imply the presence of spatial heterogeneity. Scanning statistics identified a cluster of obese outcomes centered on a grocery store that overlapped with a cluster of grocery stores in the mid-sized city in the sample. This suggests that living close to a grocery store increased the likelihood of obesity, but only in the urban subsample. The logistic regression mirrored this finding, not only in geographic location but also in sign, suggesting the presence of spatial heterogeneity.

The second objective further tests for the presence of spatial heterogeneity by testing the robustness of the findings in the logistic regression to potential existence of measurement error bias in the accessibility dimension due to intra store-type quality variation. Previous research evaluating the non-spatial dimensions of food access have found significant variation in quality between stores, even of the same type (i.e., variations in quality of supermarkets) [37,38,39]. However, typical measures of accessibility would not be sensitive to this source of variation, which can result in measurement bias if variations in quality are associated with health outcomes. To address this weakness, the analysis included results from a NEMS assessment of grocery stores that provides an objective measure of quality [40]. This variable was used to generate a quality-weighted measure of grocery store accessibility (QWGA). When the QWGA variable was used in the conditional logistic regression, the relationship between accessibility and obesity was nullified. The lack of robustness in the findings suggests that results, when a typical measure of accessibility was used, were influenced by spatial heterogeneity. This finding is similar to previous findings that identified spatial heterogeneity in a national-level food access study [28]; it also suggests the relationship between accessibility and obesity is more complicated than distance alone.

Overall, this paper has provided a method to address the spatial dimension in a food access model that utilizes the accessibility dimension. The case study results suggest that the relationship between food store accessibility and obesity may be sensitive to the empirical challenges posed by using data that contains a spatial dimension. In order to better isolate the effect of the food environment on health outcomes, future research should focus on addressing issues of spatial heterogeneity, endogeneity, and standard error bias.

There are several limitations to this analysis. First, the sample size was small, with regards to both the geographic area covered and number of survey respondents. This may limit the ability to generalize these findings to other settings and populations. Second, accessibility was only measured as the distance to the nearest grocery store, rather than the distance to a utilized grocery store. Thus, the findings and methods may not be relevant to other definitions of accessibility. Third, prior research has shown that the use of cluster robust standard errors may not be sufficient to address spatial autocorrelation [26]. However, since the research objectives focus on the interpretation and bias in the estimation of the accessibility parameter, this challenge is left to future research. The final limitation is the inability to control for the variation in quality amongst the noxious retailers.

## 5. Conclusions

The purpose of this study was to investigate the spatial dimension of food access to better understand potential reasons for the mixed findings of past research and to motivate future research. The study illustrated how spatial scanning statistics can be used to better identify sources of local spatial variation that may indicate spatial heterogeneity in a regression setting. Spatial scanning statistics detected a cluster of obesity centered on a grocery store suggesting that living closer to a grocery store increased the likelihood of obesity. Although this same relationship was also detected by a conditional logistic regression, the relationship was nullified after controlling for grocery store quality. These findings suggest that the results from studies that rely solely upon geographical measures of food access may be sensitive to the local spatial variation in the dataset and that the relationship between health and place may be more complex than distance alone.

## Figures and Tables

**Table 1:** Quality-weighted grocery store accessibility (QWGA) example. NEMS: Nutrition Environment Measures Survey.

Nearest Grocery Store				
**Household**	**Distance**	**Store NEMS Score**	**Store NEMS Rank**	**QWGA**
A	1	5	1	1
B	6	10	2	3
C	6	20	3	2
D	10	30	4	2.5

**Table 2:** Summary statistics of individual-level variables.

Summary Statistics of Individual-Level Variables	Mean (SD)		
	Full Geography	Rural	Urban
Urban (%)	38.75		
	(48.75)		
*Respondent Characteristics*			
BMI	29.01	28.21	30.25 ***
	(6.43)	(5.20)	(7.81)
Obesity (%)	38.07	34.31	43.87 **
	(48.59)	(47.52)	(49.70)
Female (%)	76.14	73.69	80.00 *
	(42.65)	(44.07)	(40.06)
Age	55.95	58.64	51.69 ***
	(17.18)	(15.62)	(18.63)
Caucasian	60.52	75.90	36.19 ***
	(48.91)	(42.81)	(48.13)
African American	34.81	19.88	58.41 ***
	(47.67)	(39.95)	(49.37)
Other or multiracial	2.71	2.41	3.17
	(16.24)	(15.35)	(17.56)
*Employment Status*			
Employed (%)	29.77	31.53	26.98
	(45.75)	(46.51)	(44.46)
Unemployed (%)	7.01	5.62	9.21
	(25.55)	(23.06)	(28.96)
Retired (%)	32.10	37.35	23.81 ***
	(46.72)	(48.42)	(42.66)
*Highest Level of Education*			
Less than high school (%)	17.36	17.30	17.46
	(37.90)	(37.87)	(38.02)
High school or General Education Dipolma (GED) (%)	35.96	36.22	35.56
	(48.02)	(48.11)	(47.94)
Some college (%)	30.79	31.19	30.16
	(46.19)	(46.37)	(45.97)
College (%)	15.89	15.29	16.83
	(36.58)	(36.03)	(37.47)
*Income Level*			
Less than $20,000 USD (%)	45.36	32.07	65.36 ***
	(49.82)	(46.73)	(47.67)
Between $20,000 and $50,000 USD (%)	33.67	43.23	19.29 ***
	(47.29)	(49.60)	(39.52)
Greater than $50,000 USD (%)	20.97	24.70	15.36 **
	(40.74)	(43.18)	(36.12)

Test for Urban vs. Rural: * = *p* < 0.05; ** = *p* < 0.01; *** = *p* < 0.001; BMI: body mass index. USD: United States Dollars.

**Table 3:** Summary statistics of food environment variables.

Summary Statistics of Food Environment Variables	Mean (SD)		
	Full Geography	Rural	Urban
Distance to nearest grocery store	3.67	5.24	1.19 ***
	(3.82)	(4.13)	(0.81)
Distance to nearest fast food restaurant	2.81	3.98	0.96 ***
	(2.91)	(3.14)	(0.83)
NEMS score of nearest grocery store	21.45	20.40	23.10 ***
	(7.84)	(8.39)	(6.58)
Noxious retailer count (10-mile radius for rural; 1-mile radius for urban)	38.98	57.99	8.92 ***
	(37.23)	(36.11)	(6.46)
Quality-weighted grocery store accessibility (QWGA)	0.48	0.73	0.10 ***
	(1.29)	(1.61)	(0.09)

Test for Urban vs. Rural: *** = *p* < 0.001.

**Table 4:** Results of scanning statistic with Poisson distribution and Bernoulli distribution.

Results of Scanning Statistic with Poisson Distribution and Bernoulli Distribution	Number of Clusters	Number of Significant Clusters	Radius (Miles)
*Poisson Distribution*			
*Grocery Stores*			
Higher than expected	10	1	0 ***
Lower than expected	2	0	
*Bernoulli distribution*			
Obesity with grocery store foci	4	1	1.48 ***

*** = *p* < 0.001.

**Table 5:** Results from conditional logistic regression of effect of food access variables on likelihood of obesity.

Results from Conditional Logistic Regression of Effect of Food Access Variables on Likelihood of Obesity	AME (CRse)	Rural	City
	Full Geography		
*Model 1*			
Grocery store accessibility	−0.003	0.003	−0.09 *
	(0.005)	(0.006)	(0.04)
*Model 2*			
Quality-weighted grocery store accessibility (QWGA)	−0.01	0.007	−0.51
	(0.01)	(0.009)	(0.41)
*Model 3*			
Quality-weighted grocery store accessibility (QWGA)	−0.01	0.002	−0.29
	(0.02)	(0.009)	(0.40)
Noxious store availability	−0.001	−0.001	0.006
	(0.001)	(0.001)	(0.004)

* = *p* < 0.05; Controls included in all models but suppressed in above results: binary indicator for sex, indicators for race (African American, Caucasian, other), continuous variable for age, indicators for highest level of education (less than high school, high school, some college, college), indicators for income (less than $20,000 USD, between $20,000 USD and $50,000 USD, greater than $50,000 USD), and indicators for employment status (employed, unemployed, retired). AME: average marginal effects. CRse: cluster robust standard errors.

## References

[1] Walker R., Keane C., Burke J. (2010). Disparities and access to healthy food in the United States: A review of food deserts literature. Health Place.

[2] Beaulac J., Kristjansson E., Cummins S. (2009). A systematic review of food deserts, 1966–2007. Prev. Chron. Dis..

[3] Aggarwal A., Cook A., Jiao J., Seguin R., Moudon A.V., Hurvitz P. (2014). Access to supermarkets and fruit and vegetable consumption. Am. J. Public Health.

[4] Dunn R., Dean W., Johnson C., Lediner A., Sharkey J. (2012). The effect of distance and cost on fruit and vegetable consumption in Rural Texas. J. Agric. Appl. Econ..

[5] Michimi A., Wimberly M. (2010). Associations of supermarket accessibility with obesity and fruit and vegetable consumption in the Conterminous United States. Int. J. Health Geogr..

[6] Capsi E.C., Sorensen G., Subramanian S.V., Kawachi I. (2012). The local food environment and diet: A systematic review. Health Place.

[7] McKinnon R.A., Reedy J., Morrissette M.A., Lytle L.A., Yaroch A.L. (2009). Measures of the food environment: A compilation of the literature, 1990–2007. Am. J. Prev. Med..

[8] Gustafson A., Hankins S., Jilcott S. (2012). Measures of the consumer food store environment: A systematic review of the evidence 2000–2011. J. Community Health.

[9] Papas A.M., Alberg A.J., Ewing R., Helzisouer K.J., Gary T.L., Klassen A.C. (2007). The built environment and obesity. Epidemiol. Rev..

[10] Charreire H., Casey R., Salze P., Simon C., Chaix B., Banos A., Badariotti D., Weber C., Oppert J. (2010). Measuring the food environment using geographical information systems: A methodological review. Public Health Nutr..

[11] Morland K. (2015). Local Food Environment Food Access in America.

[12] Powell L., Bao Y. (2009). Food Prices, Access to food outlets and child weight. Econ. Hum. Biol..

[13] Cobb L., Appel L., Franco M., Jones-Smith J., Nur A., Anderson C. (2015). The relationship of the local food environment and obesity: A systematic review of methods, study quality, and results. Obesity.

[14] Lytle L., Sokol R. (2017). Measures of the food environment: A systematic review of the field, 2007–2015. Health Place.

[15] Ver Ploeg M., Dutko P., Breneman V. (2014). Measuring food access and food deserts for policy purposes. Appl. Econ. Perspect. Policy.

[16] Cummins S., Flint E., Matthews S. (2014). New neighborhood grocery store increased awareness of food access but did not alter dietary habits or obesity. Health Aff..

[17] Gamba R., Schuchter J., Rutt C., Seto E. (2015). Measuring the food environment and its effects on obesity in the United States: A systematic review of methods and results. J. Community Health.

[18] Pearson T., Russell J., Campbell M., Baker M. (2005). Do food deserts’ influence fruit and vegetable consumption? A cross sectional study. Appetite.

[19] Sadler R., Gilliland J., Arku G. (2013). A food retail-based intervention on food security and consumption. Int. J. Environ. Res. Public Health.

[20] Wang M., MacLeod K., Steadman C., Williams L., Bowie S., Herd D., Luluquisen M., Woo M. (2007). Is the opening of a neighborhood full-service grocery store followed by a change in the food behavior of residents?. J. Hunger Environ. Nutr..

[21] Bivand R., Pebesma E., Gomez-Rubio V. (2013). Applied Spatial Data Analysis.

[22] Blitstein J., Snider J., Evans W.D. (2012). Perceptions of the food shopping environment are associated with greater consumption of fruits and vegetables. Public Health Nutr..

[23] Ball K., Timperio A., Crawford D. (2009). Neighbourhood socioeconomic inequalities in food access and affordability. Health Place.

[24] Susan C., Florax R., Snyder S., Miller C. (2010). Obesity and access to chain grocers. Econ. Geogr..

[25] Susan C., Florax R., Snyder S. (2013). Obesity and fast food in urban markets: A new approach using geo-referenced micro data. Health Econ..

[26] Thoma B., Diamond R., Imbens G., Kolesar M. (2012). Clustering, spatial correlations, and randomization inference. J. Am. Stat. Assoc..

[27] Lorna F., Clarke G., Cade J., Edwards K. (2012). Fast food and obesity. Am. J. Prev. Med..

[28] Sang-Hyun C., Grigsby-Toussaint D., Bradford N., Choi J. (2013). Can geographically weighted regression improve our contextual understanding of obesity in US? Findings from the USDA food atlas. Appl. Geogr..

[29] Kulldorff M. (1997). A spatial scan statistic. Commun. Stat. Theory Method..

[30] Amy A., Gebreab S., Mair C., Roux A.D. (2012). A review of spatial methods in epidemiology, 2000–2010. Annu. Rev. Public Health.

[31] Baker E., Schootman M., Barnidge E., Kelly C. (2006). The role of race and poverty in access to foods that enable individuals to adhere to dietary guidelines. Prev. Chronic Dis..

[32] Da P., Pearce J. (2011). Obesity-promoting food environments and the spatial clustering of food outlets around schools. Am. J. Prev. Med..

[33] Lauren F., Kleinman K., Melly S., Sharifi M., Marshall R., Block J., Cheng E., Taveras E. (2016). Effects of proximity to supermarkets on a randomized trial studying interventions for obesity. Am. J. Public Health.

[34] Ha L., Engler-Stringer R., Muhajarine N. (2016). Walkable home neighbourhood food environment and children’s overweight and obesity: Proximity, density or price?. Can. J. Public Health.

[35] Nicole W., Ma Y., Olendzki B., Procter-Gray E., Cheng J., Kane K., Ockene I., Pagoto S., Land T., Li W. (2015). Access to healthy food stores modifies effect of dietary intervention. Am. J. Prev. Med..

[36] Tara Z.Y., Laraia B., Mujahid M., Blanchard S., Warton E.M., Moffet H., Karter A. (2016). Is a reduction in distance to nearest supermarket associated with BMI change among type 2 diabetes patients?. Health Place.

[37] Daniel B., Kouba J. (2005). A comparison of the availability and affordability of a market basket in two communities in the Chicago area. Public Health Nutr..

[38] Hubley T. (2011). Assessing the proximity of healthy food options and food deserts in a rural area in Maine. Appl. Geogr..

[39] Carolyn C., Tappe K., Hillier A., Buttenheim A., Karpyn A., Glanz K. (2013). Urban Food Environments and Residents Shopping Behavior. Am. J. Prev. Med..

[40] Karen G., Sallis J., Saelens B., Frank L. (2007). Nutrition and environment measures study in stores (NEMS-S) development and evaluation. Am. J. Prev. Med..

[41] Zoellner J.M., Motley M., Wilkinson M., Jackman B., Barlow M.L., Hill J.L. (2012). Engaging the Dan River region to reduce obesity: Application of the comprehensive participatory planning and evaluation process. Fam. Community Health.

[42] Jennie H., You W., Zoellner J. (2014). Disparities in obesity among rural and urban residents in a health disparate region. BMC Public Health.

[43] The United States Census Bureau Urban and Rural Classification. https://www.census.gov/geo/reference/urban-rural.html.

[44] Clarice C., Zoellner J., Hill J. (2013). Availability of healthy food: Does block group race and income matter?. J. Hunger Environ. Nutr..

[45] Brian S., Glanz K., Sallis J., Frank L. (2007). Nutrition and Environment measures study in restaurants (NEMS-R) development and evaluation. Am. J. Prev. Med..

[46] Nicole L., Story M., Nelson M. (2009). Neighborhood environments disparities in access to healthy foods in the US. Am. J. Prev. Med..

